# The adverse outcome pathway for breast cancer: a knowledge management framework bridging biomedicine and toxicology

**DOI:** 10.1007/s12672-023-00840-x

**Published:** 2023-12-05

**Authors:** Elena von Coburg, Sebastian Dunst

**Affiliations:** 1 German Centre for the Protection of Laboratory Animals (Bf3R), Department Experimental Toxicology and ZEBET, German Federal Institute for Risk Assessment, Berlin, Germany; 2 Institute of Nutritional Science, University of Potsdam, Nuthetal, Germany

**Keywords:** Breast cancer, AOP, Estrogen receptor, Human-relevant models, Non-animal, In vitro, In silico, Biomedical research, Toxicology

## Abstract

Breast cancer is the most common cancer worldwide, with an estimated 2.3 million new cases diagnosed every year. Effective measures for cancer prevention and cancer therapy require a detailed understanding of the individual key disease mechanisms involved and their interactions at the molecular, cellular, tissue, organ, and organism level. In that regard, the rapid progress of biomedical and toxicological research in recent years now allows the pursuit of new approaches based on non-animal methods that provide greater mechanistic insight than traditional animal models and therefore facilitate the development of Adverse Outcome Pathways (AOPs) for human diseases. We performed a systematic review of the current state of published knowledge with regard to breast cancer to identify relevant key mechanisms for inclusion into breast cancer AOPs, i.e. decreased cell stiffness and decreased cell adhesion, and to concurrently map non-animal methods addressing these key events. We conclude that the broader sharing of expertise and methods between biomedical research and toxicology enabled by the AOP knowledge management framework can help to coordinate global research efforts and accelerate the transition to advanced non-animal methods, which, when combined into powerful method batteries, closely mimic human physiology and disease states without the need for animal testing.

**Supplementary Information:**

The online version contains supplementary material available at 10.1007/s12672-023-00840-x.

## Introduction

Breast cancer is the most common cancer type and the leading cause of cancer death in women [1]. Gaining a mechanistic understanding of breast cancer to prevent cancer-related deaths is therefore a common goal shared between biomedical research and toxicology. However, the enormous knowledge on breast cancer mechanisms obtained from experimental, clinical, and toxicological testings is scattered across individual research fields, calling for intensified collaborative efforts and knowledge exchange [2]. In the light of ongoing discussions surrounding the limitations of animal model systems in translating discoveries from basic research into clinical applications [3, 4], a concerted effort to develop and catalog suitable non-animal methods that provide valuable insights into cancer development and progression is crucial. This accounts especially for diseases such as breast cancer, which exhibit pronounced species differences [5, 6]. Clearly, the transition from animal studies to alternative methods requires the concomitant development of advanced strategies to combine multiple non-animal methods that address key breast cancer mechanisms on various biological levels. Here, we performed a systematic review of the current state of published knowledge on breast cancer to outline how biomedical research and toxicology could mutually benefit from an established knowledge management framework, the so-called Adverse Outcome Pathway (AOP) [7, 8].

**Fig. 1:**
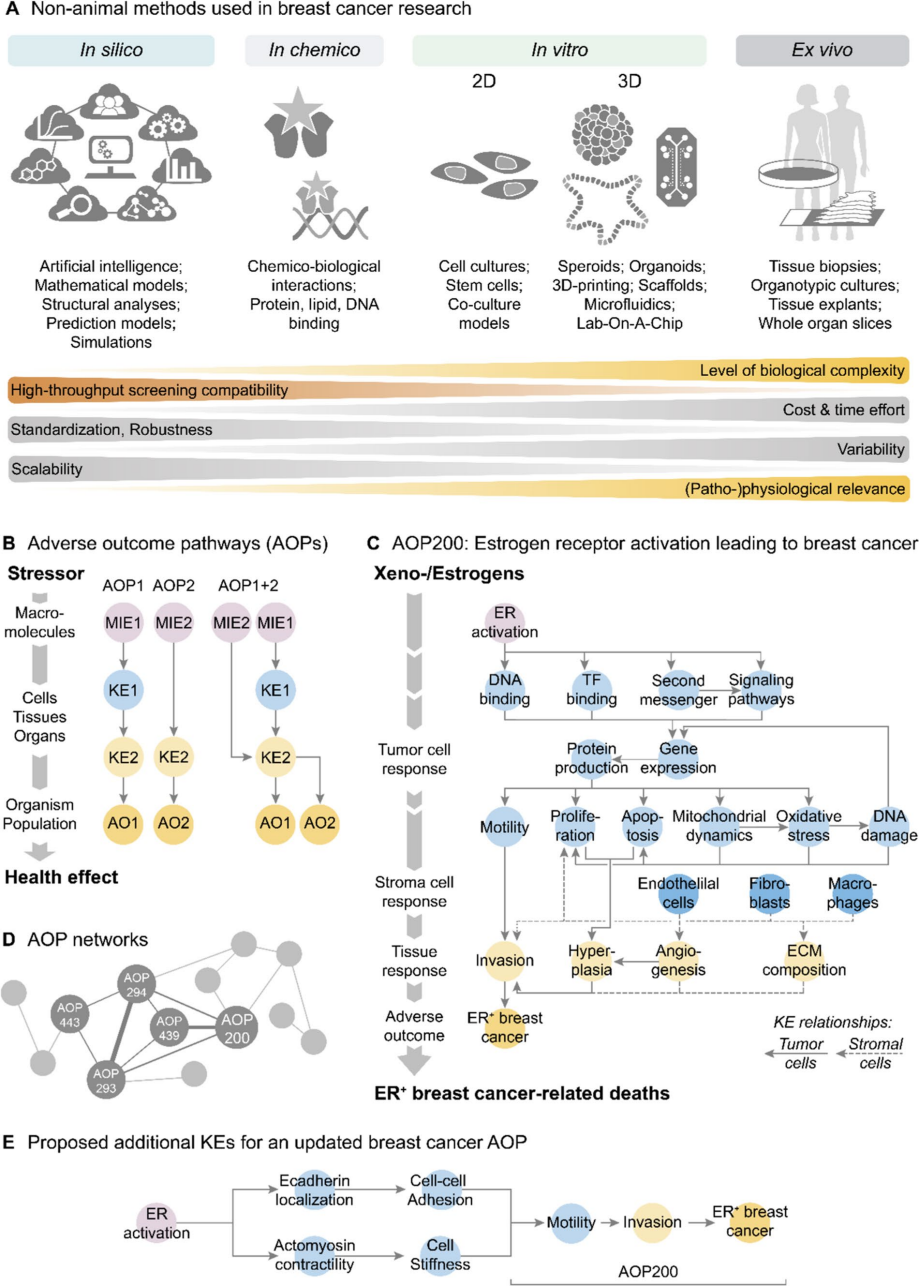
Non-animal methods and the ‘Adverse Outcome Pathway’ (AOP) concept. **A** Collection of available and emerging non-animal methods used in breast cancer research. **B** Generic structure of AOPs describing the causally-related mechanistic events that link the effect of a stressor with a specific health effect in humans. Each AOP starts with a specific molecular initiating event (MIE), and a chain of interconnected key events (KE) occurring at the cellular, tissue and organ level, which consequently lead to an adverse outcome (AO). Each event can be shared between multiple AOPs, resulting in overlapping AOPs and thus formation of AOP networks. **C** Structure of the *‘Estrogen receptor activation leading to breast cancer’* AOP (AOP 200) as proposed by [19]. The AOP summarizes the causally-related events in cancer cells (solid lines) and stromal cells (dashed lines) that lead to development of ER + breast cancer and cancer-related death upon perturbation of ER activity. **D** Representative connection (solid lines) of individual AOPs (nodes) into AOP networks (see https://aopwiki.org and Data Matrix, AOP Overview tab for more details). **E** Proposed additional KEs for an updated breast cancer AOP

National and international organizations and funding agencies provide considerable financial resources and actively engage in the development of non-animal methods that provide robust and human-relevant information at different levels of biological complexity, from molecules to cells to tissues and organs, without the need of animal testing [9–11]. These non-animal methods encompass primary cell cultures, stem cell-based approaches (iPSCs, ESCs), multi-cellular co-culture (2D, 3D), 3D-printing or scaffold-based approaches, microphysiological systems (organ/human-on-a-chip) as well as ex vivo (tissue biopsies, organotypic cultures, explants, whole organ slices), *in chemico* (binding to proteins, lipids, DNA), and in silico (artificial intelligence, modeling, simulations) methods (Fig. 1A). Many non-animal methods offer the great advantage of being compatible with high-throughput screening (HTS) approaches, which enable the rapid generation of concentration-response information for thousands of chemicals and experimental conditions, a feat unattainable with animal methods. HTS compatibility is particularly relevant in toxicology when considering the large number of chemicals marketed worldwide and countless number of combinations thereof needing evaluation for potential health risks [12]. Biomedical research faces a similar challenge in identifying efficacious drug and dosage combinations in therapeutic approaches that simultaneously modulate multiple disease-driving factors, while minimizing off-target toxicity effects [13]. However, the intended high complexity and (patho-)physiological relevance of non-animal methods comes inevitably with increased variability and decreased HTS capability (Fig. 1A), particularly when using differentiated 3D structures such as organoids to model human organ development and diseases [14, 15].

With the plethora of comprehensive research data on breast cancer and a multitude of available non-animal methods, the scientific community now faces the challenge of connecting the knowledge generated from individual studies in order to derive meaningful conclusions and to identify relevant methods and method combinations that fulfill the specific research as well as regulatory need. Importantly, the selection of relevant non-animal methods for integration into method batteries for breast cancer requires the concomitant establishment of structured knowledge management frameworks that represent the causally-related mechanistic events linked to breast cancer across different levels of biological complexity. These method batteries and knowledge management frameworks must further exhibit flexibility to quickly incorporate new scientific findings and technical progress. In the following paragraphs, we use the example of breast cancer to outline how AOPs could be used to organize the available information on breast cancer mechanisms and to facilitate the concurrent, systematic collection of non-animal methods.

## Methods and results

### A systematic review to identify relevant mechanisms and non-animal methods for inclusion into a breast cancer AOP

In an attempt to distill the extraordinary diversity of cancer into a common set of underlying core cellular parameters, the ‘Hallmarks of Cancer‘ concept was introduced [16–18]. It currently includes 14 underlying principles of cancer development that provide an actual description of the common characteristics of cancer and functional capabilities that are crucial for the ability of normal cells to form malignant tumors. However, the underlying causally-related molecular and cellular mechanisms are still actively researched. Here the AOP concept can be particularly valuable. An AOP is a hierarchical representation of a defined sequence of causally-related molecular and cellular events, whose disruption at different levels of biological organization can eventually lead to a defined adverse (pathological) outcome (AO) (Fig. 1B). For a specific adverse effect, the corresponding AOPs collate the existing information including research articles, clinical reports and public databases. To date, more than 400 AOPs (with varying levels of completeness) have already been proposed for various endpoints that are actively reviewed in a community-based approach and published online in a publicly available AOP-Wiki database (https://aopwiki.org/).

### The breast cancer AOP 200—current status

The *‘Estrogen receptor activation leading to breast cancer’* AOP (AOP 200, https://aopwiki.org/aops/200) was originally published by Morgan et al. [19] and recently expanded by Del'haye et al. [20]. It starts with perturbation of ER activity as specific molecular initiating event (MIE), followed by multiple interconnected key events (KE) at the molecular, cellular, tissue and organ level, which may eventually lead to ER + breast cancer and cancer-related death (AO) (Fig. 1C; Table 1; Data Matrix, AOP overview tab) (https://aopwiki.org/aops/200#Events). At the molecular level, KEs include causally-linked changes in gene expression and protein production of breast cells, leading on a cellular level to the escape from cell cycle regulation as well as changes in apoptosis and motility. At the tissue level, these cellular changes translate to local hyperplasia, disruption of tissue architecture, invasion, and eventually metastasis to distant organs. In addition to effects in cancer cells of the primary tumor (Fig. 1C, solid lines), the AOP further covers changes in the local tumor microenvironment (TME) (Fig. 1C, dashed lines), including endothelial proliferation, angiogenesis, and local responses from tumor-associated macrophages and fibroblasts. Importantly, these KEs align with many of the proposed ‘Hallmarks of Cancer‘ categories (Table 1; Data Matrix, AOP overview tab) and are interconnected through weighted KE relationships, which are determined based on scientific evidence (from weak to high; https://aopwiki.org/aops/200#KE_relationships).

**Table 1:** Overview of Cancer Hallmarks, the breast cancer AOP 200, and proposed breast cancer key events

Cancer hallmarks according to *hanahan (2022)*	AOP200: estrogen receptor activation leading to breast cancer				Proposed key events to be included in breast cancer-related AOPs			
Cancer Hallmark	Biological level	Event	Type	AOP-Wiki ID	Biological level	Event	Type	AOP-Wiki ID
	Prototypical stressor	Estrogen production, altered	Stressor	n.a				
		Xenobiotics, exposure	Stressor	n.a				
	Molecular	Activation, Estrogen receptor	MIE	1181	Molecular	Altered, Circadian clock	KE	n.a
	Molecular	Increased, ER binding to DNA (classical pathway)	KE	1187				
	Molecular	Increased, ER binding to T.F. to DNA (non-classical pathway)	KE	1188				
	Molecular	Altered, Gene Expression	KE	1239				
	Cellular	Increased, Second Messenger Production	KE	1242				
	Cellular	Increased, Non-genomic signaling	KE	1191				
	Cellular	Altered, Protein Production	KE	1240				
Genome instability and mutation	Molecular	Increased, Oxidative Stress	KE	1088				
	Molecular	Increase, DNA damage	KE	1194				
Nonmutational epigenetic reprogramming	-	-	-	-	-	-	-	-
Tumor-promoting inflammation	Cellular	Activation, Macrophages *	KE	1198	Cellular	Occurrence, cancer-related exosomes	KE	n.a
Polymorphic microbiomes	-	-	-	-	-	-	-	-
Sustaining proliferative signaling, Evading growth suppressors, Enabling replicative immortality	Cellular	Increase, Cell Proliferation (Epithelial Cells)	KE	1182	Cellular	Induction, breast cancer stem cells (BCSCs)	KE	n.a
	Cellular	Increased, Proliferation (Endothelial cells)	KE	1189				
	Tissue	Increased, Ductal Hyperplasia	KE	1192				
Senescent cells	-	-	-	-	-	-	-	-
Resisting cell death, Avoiding immune destruction	Cellular	N/A, Mitochondrial dysfunction 1 *	KE	177				
	Cellular	Decreased, Apoptosis (Epithelial Cells)	KE	1183				
Deregulation cellular metabolism	-	-	-	-	Cellular	Altered, metabolic activity	KE	n.a
Unlocking phenotypic plasticity	Cellular	Modulation, Extracellular Matrix Composition *	KE	1195	Cellular	Activation, cancer associated adipocytes*	KE	n.a
	Cellular	Activation, Fibroblasts *	KE	1197				
Inducing or accessing vasculature	Cellular	Increased, Angiogenesis	KE	1213	Cellular	Impairment, endothelial network	KE	110
					Organ	Occurrence, vascular Remodeling	KE	2069
					Tissue	Induction, tumor cell intra-/extravasation	KE	n.a
					Tissue	Occurrence, circulating tumor cells	KE	n.a
Activating invasion and metastasis	Cellular	Increased, motility	KE	1241	Cellular	Decreased, cell–cell adhesion (Epithelial cell)	KE	n.a
	Cellular	Increased, migration (endothelial cells)	KE	1190	Cellular	Decreased, cell stiffness (Epithelial cell)	KE	n.a
	Cellular	Increased, invasion	KE	1196				
	Adverse outcome	N/A, breast cancer	AO	1193				

*n.a*. not yet available

^*^Included in Key Event summary but Key Event Relationships not yet defined

As the number of AOPs addressing cancer-related and other disease-relevant mechanisms is constantly increasing, so is the overlap of shared MIEs, KEs or AOs among them. This will ultimately lead to the combination and interconnection of AOPs into AOP networks [21, 22], which has recently been demonstrated for endocrine-mediated perturbations [23], thyroid hormone disruption [24], and carcinogenicity [25]. The breast cancer AOP 200 likewise interconnects with three other AOPs that address different modes-of-action leading to breast cancer, i.e., aryl hydrocarbon receptor activation (AOP 439) [26], increased DNA damage (AOP 293), and increased reactive oxygen and nitrogen species (AOP 294) (Fig. 1D; Data Matrix, AOP overview tab). This way, the increasing scientific knowledge on breast cancer mechanisms could be effectively integrated in a growing catalogue of defined MIEs, KEs, and AOs linked by specified key event relationships.

### Identification of additional KEs for an updated breast cancer AOP

In order to investigate to what extent the existing MIE, KEs, and AO of the breast cancer AOP 200 are represented by published biomedical and toxicological research articles, we systematically reviewed and categorized 299 relevant publications from the PubMed database. With a particular focus on recent original studies (published within the last five years, no reviews) that used non-animal methods to study mechanisms and (environmental) stressors of breast cancer, we collected the retrieved articles and their categorizations in a Data Matrix (Data Matrix, Literature classification tab). The search strategy was based on MeSH term combinations that describe the study focus and methodologies used (Data Matrix, All queries tab), followed by a manual inclusion of high priority publications based on title and abstract review.

**Fig. 2:**
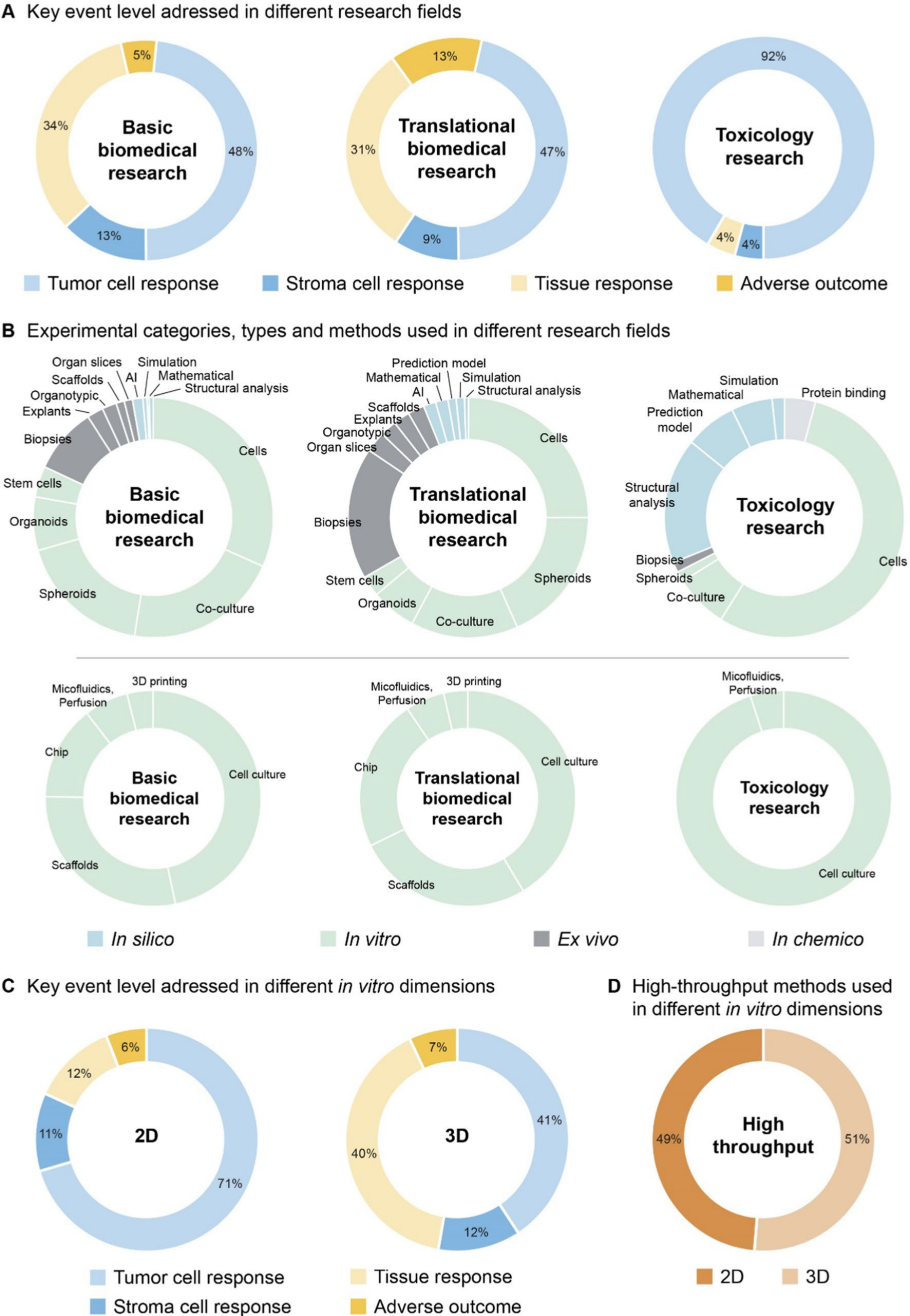
Use of non-animal breast cancer methods in biomedical research and toxicology. **A**-**D** Visualization of the collected publications and their classifications according to the addressed KE levels (tumor cell, stromal cell, tissue, adverse outcome) or the experimental categories (in silico, *in chemico*, in vitro, in vivo), experimental types (e.g. cells, biopsies, AI), in vitro methods (e.g. cell culture, scaffolds, lab-on-the-chip), in vitro dimensions (2D, 3D), and high-throughput capacity used in different research fields (basic and translational biomedical research, toxicology) (see Data Matrix, Analysis tab for more details)

We next determined the main study focus and mapped these publications to individual categories representing key breast cancer mechanisms, which included the established MIE, KEs, and AO of the breast cancer AOP 200 (Data Matrix, Literature classification tab). These 35 categories consisted of (environmental) stressors, molecular and cellular responses in both primary tumor and stromal cells, responses at the tissue level, and the AO, i.e., breast cancer. We further grouped these studies into primary research fields, i.e. basic biomedical research (mechanisms of breast cancer; 160 studies, 50%) translational biomedical research (diagnostics, (pre-)clinical testing, drugs, therapy; 103 studies, 32%) and toxicology (environmental effects; 54 studies, 17%) (Data Matrix, Analysis tab). The analyzed studies from basic and translational biomedical research covered cellular (tumor and stroma) and tissue level responses to a similar extent (Fig. 2A; Data Matrix, Analysis tab). The AO, i.e. breast cancer (covering various tumor (sub-)types), was most strongly addressed by translational biomedical studies. Notably, the analyzed toxicological studies almost exclusively investigated effects on the tumor cell level. The main KEs that were investigated in the toxicological studies included the activation of the ER, effects on gene expression and proliferation. In contrast, biomedical studies covered a much broader spectrum of more complex KEs, including apoptosis, modulation of the TME, tumor growth and invasion (Data Matrix, Analysis tab).

Based on the analyzed publications, we further identified additional key breast cancer mechanisms that were not yet established as KEs (Table 1; Data Matrix, AOP overview tab). These studies are mainly from the biomedical domain (Data Matrix, Literature classification tab) and represent key breast cancer mechanisms that should be considered for timely inclusion into AOPs. In particular, important breast cancer mechanisms that were studied in many of the analyzed publications but are not yet covered by the current breast cancer AOPs relate to the decrease of cell stiffness and cell adhesion when transforming from non-malignant to metastatic states. For example, it has been shown that estrogens determine the organization of the essential cell-cell adhesion molecule E-Cadherin at adherens junctions as well as stiffness and motility of breast cancer cells [27]. The higher mechanical elasticity and deformability of cancer cells links to a reorganization of the actomyosin cytoskeleton and strongly correlates with cell malignancy and metastatic potential [28]. In addition, metastatic sites differ between individual cancer types [29] with variations in cytoskeletal organization and stiffness of breast tumor subpopulations matching the biomechanical properties of the metastasized organs being a possible mechanical indicator explaining metastatic site preferences (organotropism) [30]. In addition to changes in biomechanical properties, invasive breast tumor cells do further show a reduced cell-cell and cell-matrix adhesion, which increases their ability to detach from the primary tumor, a process mimicking the developmental epithelial-to-mesenchymal transition (EMT) program [31], and to invade the surrounding stroma. Along this line, the Cadherin- and Integrin-family transmembrane adhesion proteins have been identified to play a major role in the transition to metastatic states in breast cancer [32, 33].

These findings emphasize the relevance of decreased cell stiffness and cell adhesion as disease mechanisms (KE) in metastatic breast cancer progression, and could be included into the existing breast cancer AOPs or used to establish new AOPs. These developments would establish connections between alterations of ER activity (MIE 1181) with metastatic breast cancer (AO 1982) through reduction of cell stiffness (new KE) by modulation of actomyosin contractility as well as reduction of cell-cell adhesion (new KE) by modulation of E-Cadherin localization. Additionally, they would consider induction of EMT (KE 1457), increased cancer cell motility (KE 1241), and increased invasion (KE 1196) (Fig. 1E) as part of the disease mechanism.

### Systematic collection of available non-animal methods in a breast cancer AOP

In addition to organizing available knowledge on cancer mechanisms, AOPs could further serve as information sources for selection of suitable non-animal methods (or combinations thereof) that adequately recapitulate particular physiological conditions and disease states. However, with regard to breast cancer research, this potential of AOPs is rather unexplored. Therefore, we further categorized the collected publications according to experimental categories, experimental types, in vitro methods, in vitro dimensions, and high-throughput capacity (Fig. 2; Data Matrix, Literature classification tab).

The collected publications encompass models such as simple biochemical and cell-based models as well as more advanced co-culture models, organoids, microfluidic lab-on-the-chip, and computational models. The main KEs that were investigated using in vitro and ex vivo methods at the tumor and stromal cell level included alterations of gene expression, increased proliferation, decreased apoptosis, and activation of tumor-associated fibroblasts. The two newly proposed KEs, i.e., decreased cell stiffness and decreased cell adhesion, were addressed by a smaller number of methods (Data Matrix, Analysis tab). At the tissue level, methods focused on modulation of ECM composition, increased tumor growth, and invasion. *In chemico* and in silico methods were mainly used to investigate mechanisms leading to activation of the ER, e.g. by xenobiotic exposure, and alterations of gene expression at the tumor cell level. In addition to the collection of available methods for individual KEs, this analysis further highlights KEs for which limited or no methods have been extracted from the retrieved publications and, thus, could be prioritized when developing non-animal methods. These method gaps include increased second messenger production, alterations of the circadian clock, occurrence of cancer-related exosomes, increased proliferation and migration of endothelial cells, induction of tumor cell intra-/extravasation, and occurrence of circulating tumor cells (Data Matrix, Analysis tab).

When comparing the experimental types performed in the three research fields, toxicology is dominated by the use of 2D cell culture methods using single cell lines (Fig. 2B; Data Matrix, Analysis tab). Single cell lines have also been used most frequently in basic and translational biomedical research, but were closely followed by co-culture or spheroid models. Although 2D cell culture remains a common in vitro model for studying tumor development and progression, the frequently observed altered growth characteristics or drug responses between 2D and 3D cell cultures have led to specific recommendations for more complex models in drug discovery [34]. Three-dimensional spheroid, organoid, scaffold-based, and microfluidic lab-on-the-chip systems currently represent the most advanced in vitro models recapitulating physiological and clinically-relevant breast cancer disease conditions to a large extent [35]. Based on our analysis, these complex non-animal methods are represented to a considerably higher extent in basic and translational biomedical research compared to toxicology (Fig. 2B; Data Matrix, Analysis tab). With regard to the investigated breast cancer KEs, the simpler 2D models were mainly used to study mechanisms at the tumor and stroma cell level, whereas 3D models were applied to study more complex responses at the tissue level and the AO (Fig. 2C; Data Matrix, Analysis tab).

Simpler 2D cell culture methods are often considered to have higher compatibility with robotic HTS applications, which can provide data for thousands of tests in little time. Interestingly, this notion does not necessarily apply to the breast cancer studies we analyzed, in which 2D- and more complex 3D-based methods were used equally in HTS-related projects (Fig. 2D; Data Matrix, Analysis tab). The majority of the analyzed HTS methods were used in toxicology and translational biomedical research (Data Matrix, Analysis tab). However, current advances in 3D culture methods promise an increased integration of 3D methods into HTS platforms in the biomedical domain [36].

Notably, translational biomedical research provided the highest proportion of published articles using ex vivo methods, particularly, cells isolated from primary material (biopsies). Other methods such as cultivation of organ-like structures derived from dissociated (organotypic) or intact, non-dissociated (explants) primary material have rather rarely been used (Fig. 2B; Data Matrix, Analysis tab). These ex vivo methods mainly focused on investigating different aspects of breast cancer at the AO level, in particular to study tumor invasion using patient-derived material in spheroid and organoid models (Data Matrix, Analysis tab).

With regard to the use of in silico methods to study breast cancer-related mechanisms, about every fourth article from the toxicological research field used computational tools, particularly structural analyses to investigate the MIE, i.e. ER activation (Fig. 2B; Data Matrix, Analysis tab). For example, the quantitative structure-activity relationship (QSAR) modeling method and molecular docking simulations are frequently used in toxicology to analyze receptor-ligand interactions, e.g., to predict ligand binding affinity of environmental stressors to ERα and, thus, to identify putative ER-mediated endocrine disrupting chemicals stimulating breast cancer [37]. The resulting data are the basis for so-called grouping and read across approaches, which are currently the most commonly applied non-animal methods for identification and characterization of chemical hazards without animal testing [38]. In the biomedical domain, structural analyses are successfully employed in the drug discovery process, e.g., to explore potential targetable sites on ERα and key structural traits to develop ERα inhibitors for breast cancer therapy [39, 40].

In recent years, artificial intelligence (AI) and computer vision have made considerable progress. AI involves machine learning (ML) and deep learning (DL) algorithms, which extract knowledge from sample data, known as training data, without being explicitly programmed. Among our dataset, AI-based models have exclusively been used in basic and translational biomedical research papers related to breast cancer (Fig. 2B; Data Matrix, Analysis tab). AI, ML, and DL become promising tools supporting or even exceeding the performance of human experts with regard to analysis [41], diagnosis [42, 43], and surgery [44] of breast cancer. For example, He et al. reported the development of a DL algorithm, which has been trained on spatial gene expression data and breast tumor morphologies in order to predicting local breast cancer biomarker expression levels directly from clinical histopathology images [41]. Another recent study has introduced the ML tool MFmap, which matches cell lines to tumor and cancer subtypes and thus can aid biomedical researchers with the selection of suitable methodologies to address their research question [45]. The rise of AI models in biomedicine has further triggered the establishment of the community-driven AIMe registry, which allows developers to easily register their AIs and helps researchers identify available AI systems suitable for their use cases [46]. In combination with advanced microscopic imaging, ML-based image analysis methodologies facilitate the automatic detection and quantification of cell morphologies. One such example is the ML-based analysis of the estrogen-dependent organization of E-Cadherin at cell-cell contacts in breast cancer cells [47], which correlates with changes in cell stiffness and cell motility of breast cancer cells [27] and which we propose as relevant KEs for an updated breast cancer AOP (see Fig. 1E). Along this line, cell morphologies are generally regarded as a holistic readout reflecting the biomechanical and physiological properties of single cells and, with regard to breast cancer, have been analyzed at high throughput in image-based phenotypic screening approaches using 2D and 3D methods in toxicology and biomedical research [48–51]. Moreover, cell morphologies can aid the differentiation of cancer from non-cancer cells and provide information on their tumorigenic and metastatic potentials [52, 53]. AI might also be important to facilitate the development of even more advanced in silico prediction tools but also of more comprehensive AOP Networks.

## Discussion

### Using AOPs to accelerate the transition to advanced, human-relevant approaches in breast cancer research

This analysis of a specific subset of the published literature on breast cancer shows that both biomedical research and toxicology could mutually benefit from using AOPs as a shared platform for a more comprehensive exchange of knowledge and non-animal methods. Sharing experimental data, in particular from HTS and -omics approaches, would significantly support the verification and update of existing AOPs [54] and gene regulatory networks [55, 56]. In addition, supplementing AOPs with quantitative data (quantitative AOPs; qAOPs) by integration of diverse types of data (physico-chemical, in silico, in vitro, in vivo) using computational approaches [57–60], could further stimulate the application of the AOP concept in toxicology and biomedical research. In that regard, the growing number of qAOPs case studies that currently become available already led to the proposal of a coherent framework for development and evaluation of qAOPs and guidance for their purpose-specific, practical application [61]. Besides the obvious benefits of sharing resources and knowledge, efforts need to be strengthened in both research fields to stimulate contributions to AOP development and to eventually put AOPs into practice.

### Stimulating and rewarding contributions to breast cancer AOP development

Systematic reviews are an important approach for constructing or updating AOPs albeit strict adherence to the original and updated PRISMA guidelines [62–64] is currently not considered as an absolute requirement. Rather, the adoption of some aspects of the systematic review process including systematic search terms and a transparent description of the literature search and selection strategy are perceived as a pragmatic approach to fast-tracking AOP development while minimizing investment of time and resources [65]. In that regard, the use of text mining tools in particular can support the efficient retrieval of relevant, published literature. As an example, the recently launched AOP-helpFinder webserver [66], which screens all PubMed abstracts for associations between stressors and biological events at various levels of biological organization (MIEs, KEs, and AOs), has been used to construct AOP 439, i.e., activation of the aryl hydrocarbon receptor leading to breast cancer [26]. In addition, elaborate text mining engines such as SMAFIRA [67] can support the retrieval of relevant non-animal methods from the PubMed database by providing a ranked list of published articles for a defined study focus. The AOP-helpFinder webserver and SMAFIRA are two domain-specific examples of text mining tools that can support the systematic review process in the context of AOP development. Other systematic review tools that could be used depending on the specific use case are collected, e.g., in The Systematic Review Toolbox (http://www.systematicreviewtools.com/), a comprehensive online catalogue of tools that support a variety of stages of the systematic review process.

To further increase the number of scientifically sound AOPs and incentivize more AOP contributions, the two scientific journals ‘*Environmental Toxicology and Chemistry*’ and ‘*Environmental and Molecular Mutagenesis*’ have pioneered the scientific peer-review of AOPs according to OECD quality standards [68] and their subsequent publication as an AOP Report [69, 70]. This currently rather formal, toxicology-oriented peer-review process needs to be opened up in the future to engage more biomedical researchers in the development of AOPs, who would ultimately benefit from a ‘systematic review-like’ publication with high citation potential.

### Putting breast cancer AOPs into practice

The assembly of single methods into method batteries to address breast cancer-related questions, e.g., on cancer mechanisms, environmental stressors, diagnosis, and treatment scenarios, is challenging. However, there are examples demonstrating the successful application of AOPs to establish suitable testing strategies. The AOP for skin sensitization [71] was the first AOP-based combination of non-animal methods into so-called Defined Approaches (DAs), which recently gained international acceptance to for regulatory decision-making [72]. Similar DAs are under development or already endorsed for other endpoints, such as the DA for serious eye damage and eye irritation [73]. Even though these endpoints are considerably less complex than the events leading to breast cancer, the current progress in this field promises further success stories towards the replacement of animal tests with human-relevant non-animal methods.

## Conclusions and outlook

The global scientific output grows exponentially and has been estimated to double every nine to 17 years with hundreds of research articles being published every day [74, 75]. Massive amounts of research and testing data on breast cancer are generated by modern technologies in biomedical sciences and toxicology. Community-driven AOPs that are designed in a user-centric fashion can support the joint analysis, interpretation, and contextualization of big data. The modular design of the breast cancer AOP facilitates the straightforward addition of new knowledge and even its combination with other AOPs based on shared KEs or AOs. Using AOPs as a blueprint to organize available mechanistic knowledge on breast cancer and human-relevant methodologies may thus help to guide the targeted development of not only toxicological testing strategies but also the formulation of biomedical research questions. More human-relevant (mechanistic) data from basic and translational biomedical research and toxicology, but, importantly, also the pharmaceutical industry, and clinics can contribute to establish and constantly update AOPs less dependent on animal data with all its problems in particular in respect to species differences.

This review article further intends to raise awareness of the opportunities of the AOP concept to support the phasing-out and replacement of animal studies, to the extent possible, with already existing and emerging human-relevant methodologies that can ensure an equal or even higher degree of protection to humans. The application of these methods in the context of AOPs has great potential to further improve the reliability and human relevance of biomedical studies. This will ultimately raise the scientific confidence in the study results and ensure translatability of biomedical research from bench to clinical practice in order to benefit patient’s health while avoiding unnecessary animal testing. However, future AOPs will need to provide more ‘quantitative’ information supporting the separation of physiological responses from disease-relevant pathological responses at the cellular level. The usability of AOPs will further essentially be determined by the level of detail that is being covered. Considering the complexity and diversity of breast cancer with regard to the different subtypes and various cell populations involved, it appears very sensible that all this information cannot be covered using a single ‘generic’ breast cancer AOP. This calls for a much greater AOP diversification with regard to breast cancer in the future.

Still, the famous quote *‘All models are wrong, but some are useful’*, coined by the British statistician George E. P. Box, is just as true in statistics as it is in biomedical research and toxicology. Any breast cancer model is just a simplification of the reality, however, the generation of human-relevant data from *‘useful’* non-animal models and the crowdsourced organization of available knowledge into *‘useful’* AOPs can help to collectively create in an interdisciplinary manner a more complete picture of the relevant mechanisms and causally related events in breast cancer and to identify knowledge and methodological gaps that need to be closed.

### Supplementary Information

**Additional file 1.** Data matrix. Categorization and analysis of collected research articles using non-animal methods that are relevant for breast cancer AOPs.

## Abbreviations

AI: Artificial intelligence
AO: Adverse outcome
AOP: Adverse outcome pathway
DA: Defined approaches
DL: Deep learning
EMT: Epithelial-to-mesenchymal transition
ESCs: Embryonic stem cells
HTS: High-throughput screening
iPSCs: Induced pluripotent stem cells
KE: Key event
MIE: Molecular initiation event
ML: Machine learning
OECD: Organization for economic cooperation and development
qAOPs: Quantitative AOPs
QSAR: Quantitative structure–activity relationship modeling
TME: Tumor microenvironment
2D/3D: 2-Dimensional/3-dimensional
